# A retrospective analysis of anti-osteoporosis medication trends among patients under 50 years old in nine major regions of China from 2016 to 2019

**DOI:** 10.7717/peerj.19187

**Published:** 2025-03-28

**Authors:** Bo Chen, Liying Chen, Zhenwei Yu, Yanting Sun, Yuzhen Wang, Chen Wang, Siqi Wang, Yan Hu, Lian-Di Kan, Liu-Cheng Li

**Affiliations:** 1 Department of Pharmacy, Sir Run Run show Hospital, Zhejiang University, Hangzhou, Zhejiang Province, China; 2 Department of Nursing, Sir Run Run Shaw Hospital, School of Medicine, Zhejiang University, Zhejiang University, Hangzhou, China

**Keywords:** Osteoporosis, Prescription, Cost, Bone, Medicine

## Abstract

**Purpose:**

This study aimed to assess the national trends in osteoporosis prescriptions among Chinese adult outpatients aged less than 50 years with osteoporosis, from 2016 to 2019.

**Patients and methods:**

Prescriptions for adult outpatients with osteoporosis from hospitals in nine major areas were extracted from the database of the Hospitals Prescription Analysis Cooperative Project. Trends in the annual prescriptions and expenditure of osteoporosis were analyzed.

**Results:**

The number of osteoporosis hospital visits showed an increasing trend year by year from 18,412 in 2016 to 23,447 in 2019 (*P* = 0.029), and the corresponding cost increased from 2,083,872.94 Chinese yuan (CNY) to 2,643,508.59 CNY in 2019 (*P* = 0.032). The result showed that the share of newer osteoporotic use of medicines increased continuously, accounting for 34.3% of prescriptions and 26.8% of expenditures in 2019. The study found that in osteoporosis hospital visits under 50 years of age, the use of medicine increased year by year. However, bone resorption inhibitors and bone formation promoters in this group did not change significantly, accounting for only small proportion.

**Conclusion:**

The development of osteoporosis prescription in this study reflected the current situation of research in China. Meanwhile, in this study, we also investigated the epidemiology of osteoporosis in China in patients under 50 years of age, for whom the incidence of osteoporosis showed an increasing trend, which reminded us to accelerate the prevention of osteoporosis.

## Introduction

Osteoporosis is the most common skeletal disease and is characterized by low bone mass, destruction of bone microarchitecture, bone fragility, and increased risk of fracture (Montagnani et al., 2022). At present, the Chinese population over the age of 60 has exceeded 210 million (approximately 15.5% of the total population), and the population over the age of 65 has exceeded 140 million (approximately 10.5% of the total population), making China the country with the largest absolute number of elderly people in the world (Qian et al., 2023; Karatoprak et al., 2012). With the aging of the population, osteoporosis becomes an important public health problem in China. Early epidemiological surveys showed that the prevalence of osteoporosis among people over the age of 50 in China was 20.7% for women and 14.4% for men, while the prevalence of osteoporosis over the age of 60 was significantly higher, especially among women (Zhao et al., 2021). It was estimated that in 2006, there were nearly 70 million osteoporosis patients in China (Zhong et al., 2022), and more than 200 million people had osteopenia. Despite the lack of recent epidemiological data, the estimated number of people with osteoporosis and osteopenia in China has exceeded nearly 70 million.

At present, most of the studies have been concentrated on osteoporosis in menopausal women and elderly individuals. However, less attention has been paid to the prevalence of osteoporosis in individuals under 50 years old, and there was was still a lack of studies on osteoporosis in individuals under 50 years old (Yu & Xia, 2019). Currently, the incidence of osteoporosis is increasingly high, with more younger patients diagnosed annually, showing a trend of younger age. Moreover, the development of osteoporosis will lead to severe clinical outcomes. For example, osteoporosis increases the risk of fracture, and loss of function at the fracture site increases disability and mortality, bringing a great economic burden to the society (Wang et al., 2009). Therefore, early prevention and appropriate management are necessary.

This study focused on investigating the medicine application of osteoporosis in patients under 50 years old, aiming at providing additional evidence for the current situation of osteoporosis in China. Osteoporosis is also closely related to osteoarthritis, which requires early prevention. The therapy and management of osteoporosis in individuals under the age of 50 were retrospectively examined in this study, together with demographic information like medical prescriptions, in nine major areas. More efforts should be made to increase people’s awareness and knowledge regarding osteoporosis and to promote good bone health (Shim et al., 2020; Yu & Xia, 2019; Chen, Li & Hu, 2016).

## Materials and Methods

Portions of this text were previously published as part of a preprint (https://www.researchsquare.com/article/rs-3327853/v1).

### Ethics

This multicenter and cross-sectional study was in line with the guidelines of the World Medical Association and the Declaration of Helsinki. The Ethics Committee of Sir Run Run Shaw Hospital had reviewed the study protocol and confirmed that formal ethics approval was not required because this study only involved prescription data from the database of the Hospital Prescription Cooperation Project, which was widely used as described from previous studies. Informed consent was also waived by the Ethics Committee (Approval number, 2022-0359). Permission was obtained to access the database and all the data used was identified upon data collection.

### Study design

This study was designed as a cross-sectional study based on prescription data.

### Data source and study sample

The prescriptions were extracted from the database of the Hospital Prescription Analysis Cooperative Project, which was used in our previous studies and many other Chinese epidemiological studies (Qian et al., 2023). The database contains prescription information from participating hospitals on 40 random days each year. Prescriptions that met the following criteria were included: (1) issued for outpatients aged > 18 years and aged ≤ 50 years; (2) issued for patients with a diagnosis of osteoporosis (regardless of diagnostic criteria, type, or severity); (3) containing at least one osteoporosis (initial prescription or renewal) and issued between 2016 and 2019; and (4) from hospitals in Beijing, Chengdu, Guangzhou, Harbin, Hangzhou, Shanghai, Shenyang, Tianjin, Zhengzhou, which participated in the program continuously from 2016 to 2019. The following items were extracted from the included prescriptions: prescription code, sex, age of the patient, year issued, location, hospital code, diagnosis, generic medicine names, and cost of each medicine. This retrospective study was performed according to the guidelines of the World Medical Association and the Declaration of Helsinki. Based on that study, The Ethics Committee of the Institutional Review Board of our hospital approved the present study. In this study, 83,353 cases of osteoporosis hospital visits were initially collected. After the following inclusion and exclusion criteria were implemented.

### Assessment of medicine use

In this study, prescriptions were separated according to the medicines used for the treatment of osteoporosis and their accompanying disorders. The following types of medicine were used for osteoporosis treatment. (1) Bone mineralization promoter; calcium, which included calcium acetate, calcium carbonate, calcium citrate, calcium chloride, oyster calcium carbonate, calcium gluconate. Vitamin D and its active metabolites, which included alfacalcitol, calcitriol, paricalcitol, vitamin AD, vitamin D3, Vitamin D2. (2) Bone resorption inhibitors, bisphosphonate, which included alendronate sodium and risedronate sodium. Calcitonins, which included calcitonin and elcatonin. Estrogen, which included estrogen, estriol, estradiol and raloxifene. (3) Bone formation promoter, which included, teriparatide, recombinant teriparatide and tetrahydromethylnaphthoquinone; (4) combination medicine, which included alendronate sodium D3 and calcium carbonate D3.

Medicine usage was assessed by prescription numbers, irrespective of whether it was new or a refill. The cost was calculated by adding the price of all analyzed medicine in Chinese yuan (CNY). Trends in yearly prescriptions and costs were analyzed and further stratified by sex, age, medicine class, and specific medicine.

### Data analysis

The prescription data were processed using Microsoft Access software and exported to Microsoft Office Excel 2007 (Microsoft Corp., Redmond, WA, USA) for statistical analysis. Statistically significant differences (*P* < 0.05) were determined using SPSS 22.0 (IBM Corp., Armonk, NY, USA) by Pearson correlation coefficient.

## Results

### Number of hospital visits

**Figure 1:**
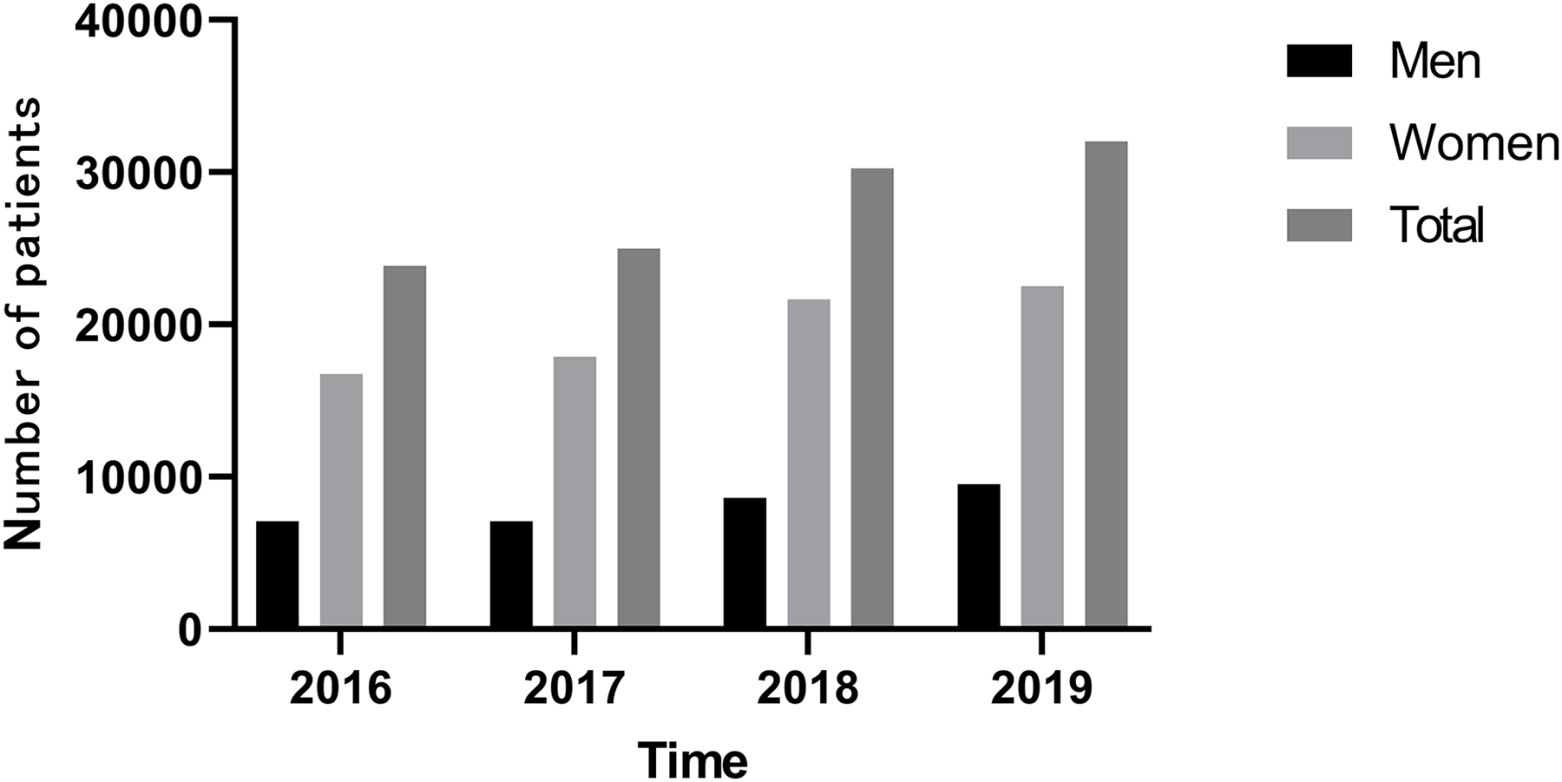
Trends in hospital visits from 2016 to 2019.

Figure 1 shows that the number of osteoporosis hospital visits showed an increasing trend year by year from 18,412 in 2016 to 23,447 in 2019 (*P* = 0.029). The percentage of man is 0.297 and woman is 0.703 in 2016 and 2019, and the percentage of man is 0.284 and woman is 0.716 in 2017 and 2018. There were all kinds of medicines in osteoporosis which included bone mineralization promoter, bone resorption inhibitors, bone formation promoter, and combination medicine in Table 1. The number of hospital visits in the 40–50 age group increased significantly (*P* = 0.05), the number of people in the 30–40 age group also increased (*P* = 0.076), and the number of people younger than 30 years old increased from 2,674 in 2016 to 2,906 in 2019, indicating that the incidence of osteoporosis is occurring at a younger age and needs to be prevented in time (Table 1).

**Table 1:** Percentage of osteoporosis medicine use in different age groups.

	Below 30	30–40	40–50	Total
2016	2,674 (0.145)	5,358 (0.291)	10,380 (0.564)	18,412
2017	2,702 (0.143)	5,401 (0.285)	10,840 (0.572)	18,943
2018	2,571 (0.114)	6,629 (0.294)	13,351 (0.592)	22,551
2019	2,906 (0.124) (*P* = 0.480)	6,781 (0.289) (*P* = 0.076)	13,760 (0.587) (*P* = 0.05)	23,447 (*P* = 0.029)

### Comparison of gender and age composition

The number of men and women showed an upward trend. The number of men increased by 23.88% from 5,493 in 2016 to 6,805 in 2019 (*P* = 0.052), and the number of women increased by 28.88% from 12,919 to 16,642 in 2019 (*P* = 0.049). The increase was higher for women than for men (Table 2).

**Table 2:** Number of prescriptions between men and women.

	2016	2017	2018	2019
Prescriptions of man	5,493 (0.298)	5,494 (0.290)	6,337 (0.281)	6,805 (0.290) (*P* = 0.052)
Prescriptions of woman	12,919 (0.702)	13,460 (0.710)	16,204 (0.719)	16,642 (0.710) (*P* = 0.049)
Total	18,412	18,954	22,541	23,447

### Comparison of medicine using in nine major areas of China

The use of osteoporosis medicine increased significantly in most cities. Compared with 2016, the use of medicine increased in all cities in 2019, among which Chengdu (*P* = 0.009), Harbin (*P* = 0.02), Hangzhou (*P* = 0.048), Shanghai (*P* = 0.028), Shenyang (*P* = 0.046) and Zhengzhou (*P* = 0.068) showed an increasing trend year by year. This shows that not only in general but also in specific cities, osteoporosis medicine use is consistent with the trend of increasing year after year (Table 3).

**Table 3:** Trends in osteoporosis medicine use in different cities by year.

	2016	2017	2018	2019
Beijing	11,236 (0.471)	11,628 (0.463)	12,900 (0.426)	11,756 (0.367) (*P* = 0.489)
Chengdu	2,327 (0.098)	3,005 (0.120)	4,500 (0.149)	5,599 (0.175) (*P* = 0.009)
Guangzhou	2,425 (0.102)	2,264 (0.090)	2,092 (0.069)	2,428 (0.076) (*P* = 0.868)
Harbin	459 (0.019)	575 (0.023)	680 (0.022)	768 (0.024) (*P* = 0.02)
Hangzhou	2,776 (0.116)	3,160 (0.126)	3,710 (0.123)	3,721 (0.1) (*P* = 0.048)
Shanghai	1,515 (0.064)	1,797 (0.072)	2,181 (0.072)	2,253 (0.070) (*P* = 0.028)
Shenyang	94 (0.004)	104 (0.004)	260 (0.009)	408 (0.013) (*P* = 0.046)
Tianjin	2,941 (0.123)	2,512 (0.100)	3,853 (0.127)	5,016 (0.157) (*P* = 0.119)
Zhengzhou	65 (0.003)	74 (0.003)	85 (0.003)	84 (0.003) (*P* = 0.068)
Total	23,838	25,119	30,261	32,033

### Cost analysis

**Figure 2:**
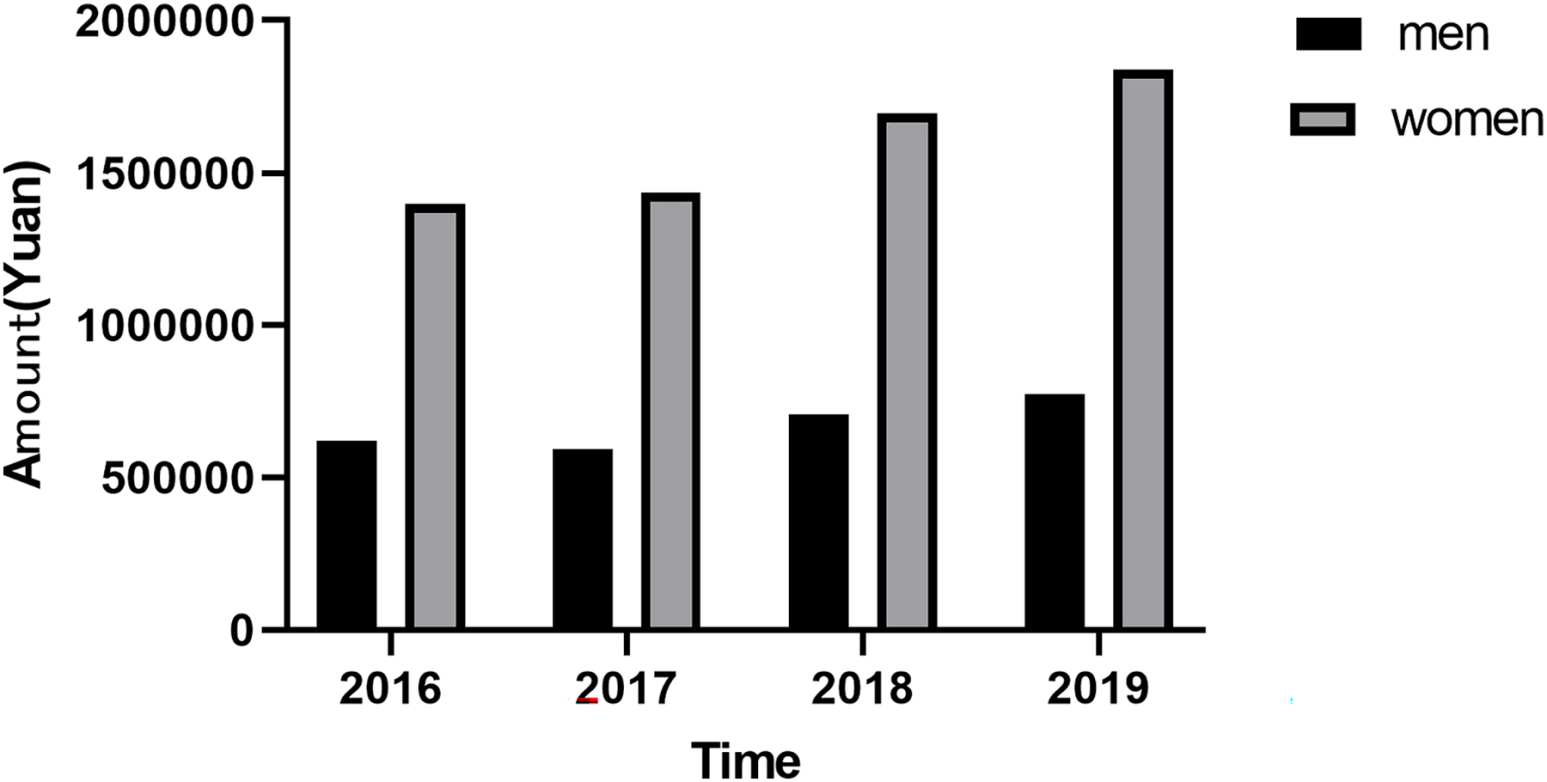
Trend of costs between men and women from 2016 to 2019.

The amount of medicine for osteoporosis is increasing year by year, and there is an upward trend among both men and women. And the total amount of money increased by 26.8%, from 2,083,872.94 CNY in 2016 to 2,643,508.59 CNY in 2019 (*P* = 0.032). The total amount of money for men increased by 24.6%, from 619,764.49 CNY in 2016 to 772,788.63 CNY in 2019 (*P* = 0.098). The total amount for women increased by 31.6 percent from 13,96,728.93 CNY in 2016 to 1,838,912.09 CNY in 2019. The increase was more dramatic for women than for men (Fig. 2).

### Summary of different types of medicine use

**Figure 3:**
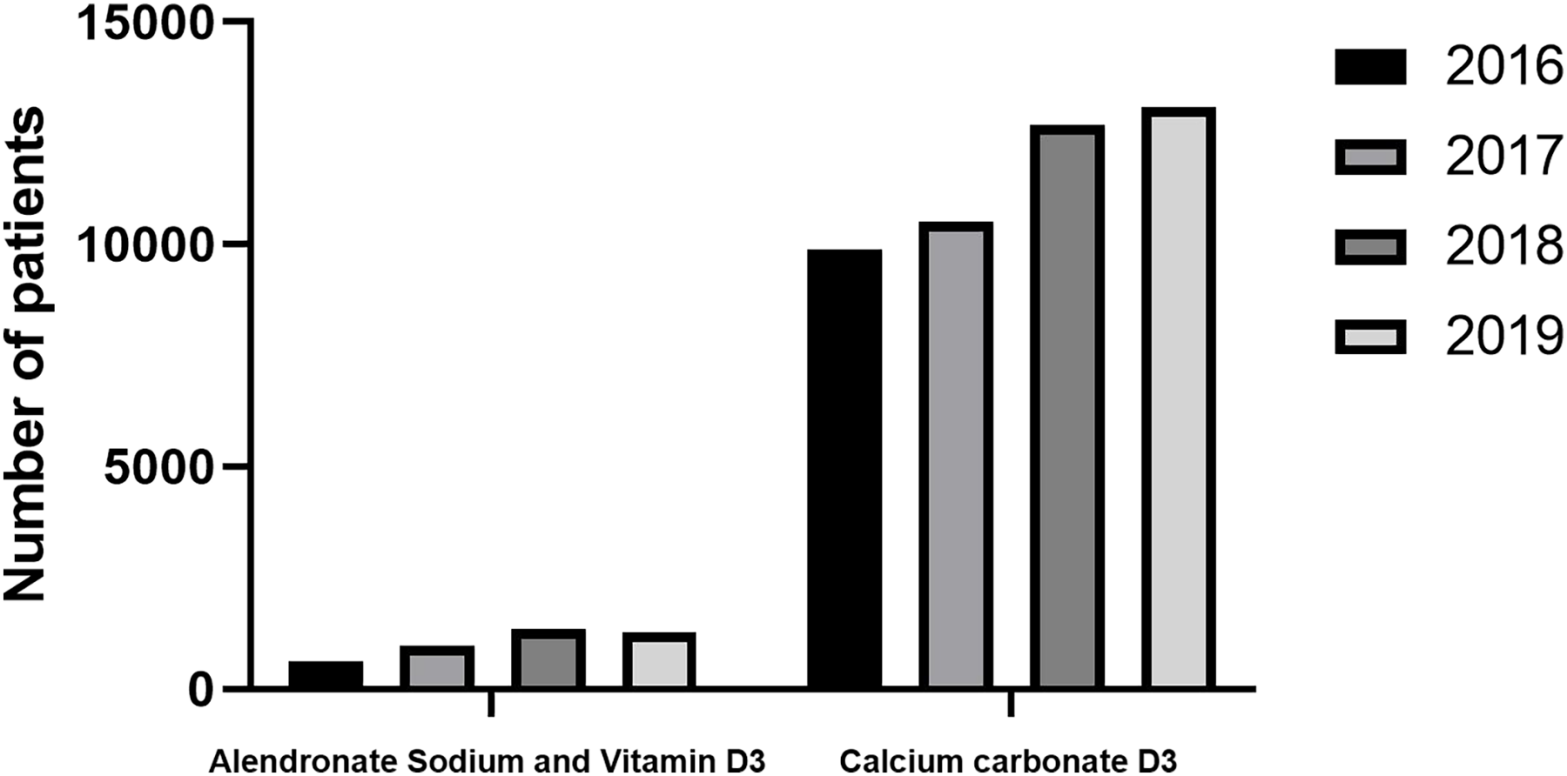
Trends of medicine used from 2016 to 2019.

In osteoporosis hospital visits for patients aged less than 50 years old, bone mineralization promoters and combination medicine preparations are still the main ones, and the bone mineralization promoters are mainly vitamin D active metabolites, while the proportion of traditional single calcium preparations is low, and there is no significant increase. It indicates that clinicians do not favor a single calcium preparation, as the clinical effect of a single calcium preparation is not significant, while the main compound of calcium carbonate D3 and alendronate D3 shows a significant clinical effect (*p* = 0.038). Calcium carbonate D3 (*p* = 0.038) was the predominant medicine in the composite, and both calcium carbonate and vitamin D3 were promoters of bone mineralization, indicating that in hospital visits by patients under 50 years of age, the primary prophylactic use was still with promoters of bone mineralization, rather than bone resorption inhibitors used in severe osteoporosis (Fig. 3 and Table 4). The high use of combination medicine preparations indicates that clinicians prefer to use combination medicines, indicating that combination medicine preparations can better promote treatment and reduce the probability of missed medication.

**Table 4:** The proportion of different kinds of medicine used in osteoporosis patients.

	2016	2017	2018	2019
Summary of promoters of bone mineralization	10,997 (0.461)	11,498 (0.458)	14,186 (0.469)	15,655 (0.489) (*P* = 0.028)
Summary of bone resorption inhibitors	2,319 (0.097)	2,016 (0.080)	2,006 (0.066)	1,942 (0.061) (P=0.127)
Bone formation promoter	15 (0.001)	23 (0.001)	37 (0.001)	45 (0.001) (*P* = 0.007)
Combination medicine	10,507 (0.441)	11,582 (0.461)	14,032 (0.464)	1,4391 (0.449) (*P* = 0.035)

## Discussion

In 2001, the National Institutes of Health (NIH) defined osteoporosis as a bone disorder characterized by decreased bone strength and increased risk of fracture, suggesting that decreased bone mass was a major risk factor for osteoporotic fractures (NIH Consensus Development Panel on Osteoporosis Prevention, Diagnosis, and Therapy, 2001). Osteoporosis can occur at any age but is more common in postmenopausal women and older men. Postmenopausal osteoporosis generally occurs in women within 5 to 10 years after menopause, and the age of menopause is generally between 45 and 55 years (Claser & Kaplan, 1997). So postmenopausal osteoporosis generally occurs after the age of 50, and senile osteoporosis generally refers to the onset of osteoporosis after the age of 70. The current research on osteoporosis focuses on these two partsaspects. There is a relative lack of research on osteoporosis before the age of 50, but osteoporosis can lead to a series of serious clinical outcomes, both in economics and in health (Blumstein et al., 2018). This study used a large database to investigate trends in osteoporosis medication use among hospital visits by patients under 50 years of age in China during 2016 to 2019.

The number of osteoporosis hospital visits showed an increasing trend year by year from 18,412 in 2016 to 23,447 in 2019 (*P* = 0.029). The number of men and women showed an upward trend. The amount of medicine for osteoporosis increased annually from 2016 to 2019, and there is an upward trend among both men and women. In osteoporosis hospital visits by patients under 50 years of age, bone mineralization promoters and combination medicine are still the main medications, and the bone mineralization promoters are mainly vitamin D active metabolites. The primary prophylactic use was still promoters of bone mineralization, rather than bone resorption inhibitors used in severe osteoporosis. The incidence of osteoporosis in China is rising among young people under the age of 50. Current studies found that the incidence of osteoporosis and the amount of medicine used in the group under the age of 50 were on the rise, which reminded us that the incidence of osteoporosis is on the rise in the young population, reminding us that we should take early actions to prevent osteoporosis, accept regular bone density examinations and timely supplementation of vitamin D3 and calcium. Bone strength is the key to maintaining human health. The prevention and treatment of osteoporosis should run through the whole life process. Osteoporotic fractures increase the disability rate and mortality rate. Therefore, the prevention and treatment of osteoporosis were equally important. The main goals of the prevention and treatment of osteoporosis include improving bone growth and development, promoting ideal peak bone mass in adulthood, maintaining bone mass and bone quality, preventing age-related bone loss and avoiding falls and fractures (Yu et al., 2021; Yong & Logan, 2021; Adami et al., 2021; Johnson et al., 2021; Ma et al., 2023).

Nevertheless, this study had some limitations. In addition, Chinese medicine has been proved to have a significant effect on osteoporosis in some studies (Wang et al., 2017; He et al., 2019) Due to the lack of traditional Chinese medicine related data collection in this database, some patients may take traditional Chinese medicine at the same time, which will have a certain impact on the accuracy of conclusion. In the later stage, we will strive to further improve the database. Otherwise, prescription data were obtained from hosipital in nine major areas of China that participated in the Hospital Prescription Analysis Cooperative Project, which may have resulted in sampling bias as these data may not accurately represent the whole of China. We analyzed prescription information for outpatient osteoporosis but could not confirm whether patients took their medications as prescribed. And we failed to give the estimates of osteopenia and osteoporosis and the types of osteoporosis (primary or secondary) in those hospitals where data was taken for prescriptions.

Our data were also obtained using random sampling; hence, some information of drug combinations might have been omitted, and evaluating the continuous medication changes in patients with osteoporosis was difficult. Future studies will need to evaluate real-world changes in anti-osteoporosis medications by continuous follow-up of patients with osteoporosis. The prescription data of patients with osteoporosis in different regions of China must be collected to analyze the situation of pharmacological treatments for osteoporosis in the whole of China.

## Supplemental Information

10.7717/peerj.19187/supp-1 Supplemental Information 1 Raw data 1.

10.7717/peerj.19187/supp-2 Supplemental Information 2 Raw data 2.

10.7717/peerj.19187/supp-3 Supplemental Information 3 Codebook.

## Additional Information and Declarations

### Data Availability

The following information was supplied regarding data availability:

The raw data is available in the Supplemental Files.
