# Supplementary material for: A retrospective analysis of anti-osteoporosis medication trends among patients under 50 years old in nine major regions of China from 2016 to 2019
Source: PeerJ. 2025 Mar 28;13:e19187. doi: 10.7717/peerj.19187 (PMC11956766; doi:10.7717/peerj.19187)
Supplement: Supplemental Information 3 [file peerj-13-19187-s003.docx]

北京：Beijing上海：Shanghai杭州：Hangzhou广州：Guangzhou哈尔滨：Harbin沈阳：Shenyang

天津：Tianjin郑州：Zhengzhou成都：Chengdu

Beijing(北京)，Shanghai(上海)，Hangzhou(杭州)，Guangzhou(广州)，Harbin(哈尔滨)，Shenyang(沈阳)，Tianjin(天津)，Zhengzhou(郑州)，Chengdu(成都) are nine major cities of China.

门诊：outpatient department

男：man 女：woman

calcium acetate（牡蛎酸钙），calcium carbonate（碳酸钙），calcium citrate（枸橼酸钙），

calcium chloride（氯化钙），oyster calcium carbonate（牡蛎碳酸钙），calcium gluconate（葡萄糖酸钙），gluconate（葡萄糖酸钙），alfacalcitol（阿法骨化醇）， calcitriol（骨化三醇）， calcitriol（帕立骨化醇）， vitamin AD（维生素AD），vitamin D3（维生素D3），Vitamin D2（维生素D2）,alendronate sodium （阿仑膦酸钠），risedronate sodium（利塞磷酸钠），Calcitonin（降钙素），estrogen（雌激素），estriol（雌三醇），estradiol （雌二醇），raloxifene（雷诺昔芬），teriparatide（特立帕肽），recombinant teriparatide（重组特立帕肽），tetrahydromethylnaphthoquinone（四氢甲基萘醌），alendronate sodium D3（阿仑膦酸钠D3），calcium carbonate D3（碳酸钙D3）

骨质疏松（osteoporosis）

高胆固醇血症（hypercholesteremia），动脉粥样硬化（atherosclerosis），高血压（hypertension），冠心病（coronary heart disease），便秘（constipation），脑血管病（cerebrovascular disease），糖尿病（diabetes），结膜炎（ophthalmia），肾虚证（kidney deficiency syndrome），前列腺肥大（Hypertrophy of prostate），白内障（cataract），类风湿性关节炎（rheumatoid arthritis），脑血管病（blood vessel of brain），上呼吸道感染（upper respiratory infection），口腔粘膜溃疡（oral ulcer），眼底动脉硬化（Fundus arteriosclerosis），下肢动脉粥样硬化闭塞症（Atherosclerotic obliterans of the lower extremity），哮喘（asthma），慢性肾功能不全（chronic renal insufficiency），高尿酸血症（hyperuricemia），维生素缺乏（hypovitaminosis），腹胀（ventosity），神经炎（neuritis），反流性食管炎（reflux esophagitis），肝硬化（liver cirrhosis），足癣（tinea pedis），失眠（insomnia），咳嗽（cough），慢性肾脏病（chronic kidney diseases），肝损害（liver damage），腰椎管狭窄（lumbar spinal stenosis），抑郁状态（depressive state），焦虑状态（anxiety state），肠道菌群失调（alteration of intestinal flora），甲状腺结节（thyroid nodule），干眼症（xerophthalmia），肺纤维化（pulmonary fibrosis），肌筋膜炎（myofascitis），慢性肾小球肾炎（chronic glomerulonephritis），糖尿病性周围神经病（Diabetic peripheral neuropathy），心律失常（arrhythmia），韦伯综合征（Weber syndrome），发热（fever），炎症性肠病（inflammatory bowel disease），白塞病（behcet disease），狼疮性肾炎（lupus nephritis），肌炎（myositis），动脉栓塞（arterial embolism），甲状腺功能减退（hypothyroidism），口角炎（angular cheilitis），记忆力下降（memory deterioration）
